# Supplementary material for: Preventive Counseling in Routine Prenatal Care—A Qualitative Study of Pregnant Women’s Perspectives on a Lifestyle Intervention, Contrasted with the Experiences of Healthcare Providers
Source: Int J Environ Res Public Health. 2022 May 18;19(10):6122. doi: 10.3390/ijerph19106122 (PMC9140722; doi:10.3390/ijerph19106122)
Supplement: Supplementary file 1 [file ijerph-19-06122-s001.zip › Supplementary Tables.pdf]

International Journal of
Environmental Research and Public Health

MDPI

Table S1. Topics of the interview guide*: Interviews with pregnant women.

Topic	Question
Icebreaker-Question	Can you please tell me about your last (GeMuKi-)counseling session at the gynecologist's practice? You are welcome to report the entire process from entering to leaving the practice.
Procedure of the counseling	Can you please tell me what you liked about the counseling?. Would you prefer anything to be done differently?
	Can you please tell me about the (GeMuKi-)counseling session with your midwife?
Content of the counseling sessions	Can you tell me more about the content of the counseling sessions?
	Can you describe what you have changed about your lifestyle since you got pregnant?
Digital support (GeMuKi-App)	Can you please describe your use of the app?
	What do you like and/or dislike about the app?
Interprofessional collaboration	Can you please describe the cooperation between your gynecologist and your midwife?
	What is your general opinion on multi-professional healthcare providers undertaking lifestyle counseling?
	How have the medical assistants been involved in the counseling sessions?
	Have you had any contact with other health professionals (e.g., psychologists, nutritionists, lactation consultants, etc.) during pregnancy? If so, can you tell me more about the occasion and your experience with the contact?
Needs and Expectations	What do you expect from (lifestyle-) counseling during pregnancy?
	On what topics and in what context would you like to receive counseling?
Satisfaction	Considering all things, how satisfied are you with your participation in the GeMuKi-project?
Closing-Question	Is there anything important we haven't discussed yet that you would like to add?

* The interviews were conducted in German. The interview guides were translated for publication by two researchers.

Table S2 Topics of the interview guide*: Interviews with healthcare providers

Topic	Question
Icebreaker-Question	Can you please tell me about your last counseling session with a participant of the GeMuKi intervention? (Please describe the entire process.)
Implementation into practice routines	Can you please describe the launch of the intervention in your practice?
	How well does preventive counseling fit with the daily practice routines?
	How did the intervention change your counseling during pregnancy?
Adoption and Acceptance by patients	How do women respond to the additional preventive counseling?
	How do women with low socio-economic status respond to the additional counselling?
Digital support	Can you please describe your work with the counseling tool “GeMuKi-Assist”?
	What do you like or dislike about the counseling tool?
Interprofessional collaboration	Can you please describe what has changed about your practice's collaboration with midwives because of the project?
	How do your colleagues in the region react to the project?
Implementation into standard care	What needs to be done in order to successfully implement the intervention into routine prenatal care?
	What do you need in your practice in order to take the additional time needed for the counseling?
	What needs to be done in order to improve prenatal care in general?
Closing-Question	Is there anything important we haven't discussed yet that you would like to add?

* The interviews were conducted in German. The interview guides were translated for publication by two researchers.
